# Biomedical Research and Corporate Interests: A Question of Academic Freedom

**DOI:** 10.4103/0973-1229.37086

**Published:** 2008

**Authors:** Leemon McHenry

**Affiliations:** * *Lecturer in Philosophy, California State University, Northridge, USA*

**Keywords:** Academic freedom, Biomedical research, Critical rationalism, Ghostwriting, Key opinion leaders, Pharmaceutical funding, Pharmaceutical industry

## Abstract

The current situation in medicine has been described as a crisis of credibility, as the profit motive of industry has taken control of clinical trials and the dissemination of data. Pharmaceutical companies maintain a stranglehold over the content of medical journals in three ways: (1) by ghostwriting articles that bias the results of clinical trials, (2) by the sheer economic power they exert on journals due to the purchase of drug advertisements and journal reprints, and (3) by the threat of legal action against those researchers who seek to correct the misrepresentation of study results. This paper argues that Karl Popper's critical rationalism provides a corrective to the failure of academic freedom in biomedical research.

## Introduction

Philosopher of science, Karl Popper, in *The Open Society and Its Enemies*, depicts the totalitarian, closed society as a rigidly ordered state in which individual liberty, freedom of expression, and discussion of crucial issues are ruthlessly suppressed (Popper, 1945). In place of irrational dogma and taboo, the open society, by contrast, tolerates a diversity of views, uncertainty in the fundamental questions, and values the freedom to advance ideas and have them rigorously criticized. Popper argued that rationality and science flourish in the open society. The question, however, is whether we actually have anything close to Popper's ideal of intellectual advance, especially in academic medicine. In this article, I shall present evidence that we do not, for we merely pay lip service to the free, democratic society while, in fact, the role that corporate interests play in government and in the control of academic medicine has a stifling effect on real freedom in the marketplace of ideas.

Noam Chomsky recognized that there is great merit in the hard-won freedom of speech in democracies such as the United States, in spite of the general tendency of the powers that be to manufacture consent and suppress opposition (Herman and Chomsky, 1988; Fava, 2002). A well-rounded and balanced account by media will always be marginalized by the profit motive. This, in my view, is essentially what has happened in academic medicine. Instead of a propaganda model of mass media we have a propaganda model of medical research: drug promotion masquerades as scientific research, as pharmaceutical giants have infiltrated the leading peer-reviewed medical journals, medical education, and professional conferences. A number of these companies, such as GlaxoSmithKline, Bayer, Pfizer, Bristol-Myers Squibb, AstraZeneca, Schering-Plough, Abbott Labs, TAP Pharmaceuticals, Wyeth, and Merck, have paid millions of dollars each as compensation in the last few years for problems with their products (Singh and Singh, 2005). While this might deter some of their activities, it is more likely to increase their aggressive measures to stifle opposition and manufacture consent.

## Crisis of Credibility in Biomedical Research

### Medical Communications and Ghostwriting

In the case of the medical literature, it is now fairly well known in academic medicine that pharmaceutical companies launder their promotional efforts through medical communication companies that ghostwrite articles and then pay “key opinion leaders,” chosen for their influence on prescribing physicians, to affix their signatures to the fraudulent articles (Elliott, 2004). This, in part, has led to what Fava has called “the crisis of credibility” (Fava, 2006). In the United States alone, there were 182 medical communication companies identified in a survey completed in 2001 (Golden *et al*., 2002). Some of the most frequently used medical communications companies or public relations firms include Scientific Therapeutics Information, Inc., Compete Healthcare Communications, Current Medical Directions, Complete Medical Communications Limited, Ruder Finn, Belsito and Company, and Cohn and Wolfe. Pharmaceutical companies also engage public relations firms to orchestrate campaigns against doctors who have been identified as critics of their ineffective or unsafe drugs. What is less clear, however, is the fine detail of the business, which is only just starting to emerge as a result of litigation (Kesselheim and Avorn, 2007). First, the pharmaceutical companies seeking to “launch” a new drug on the market, or to promote a new indication for a drug approved for another indication (e.g., adolescent depression, social anxiety disorder, erectile dysfunction, high cholesterol), will hire a public relations firm and medical communication company as part of their marketing strategy. Such firms will set up advisory board meetings with key opinion leaders and the marketing division of the pharmaceutical company in advance of the clinical trials. Once a trial is complete, a medical writer who is employed by the medical communications firm produces a draft of a manuscript from the summary of the Final Study Report of the clinical trial and then seeks feedback from the external ‘authors’ and the internal scientists who work within the sponsor company. The medical writer revises the draft a number of times, replies to feedback from the peer-review process and then, post-publication, replies to criticism in the letters to the editor of the journal in which the article is placed. Once the article is submitted, the medical writer disappears or is only acknowledged in the fine print for “editorial assistance.” Ghosts, after all, should remain invisible (Elliott, 2004).

According to the information revealed in the process of discovery following lawsuits, medical communication companies charge as much as $20,000 to $40,000 per article. They are, however, never acknowledged in the final publications. This sort of technical write-up, outsourced to the profit-based companies, is the most common method; its ethics subject to dispute among medical organizations. The most flagrant and unambiguous instances of ghostwriting involve drug or medical device promotion, in which an academic “author” is paid to put his or her signature to an article, although he or she has had no role at all in the research or writing of the piece. The academics who have taken the bait for these projects never reveal their ornamental function in such promotional pieces; it is only when one of them refuses to take part and then discovers the piece published under another name that we come to hear of the fraud (Fugh-Berman, 2005).

In another approach, the pharmaceutical companies lure physicians into ghostwriting projects by creating programmes designed to enhance the profile of their drugs and form bonds of loyalty with prescribing physicians by providing them with publishing opportunities. This is mainly accomplished by drug sales representatives who pitch the programmes to the physicians. Once the physicians have been recruited into the company's “publications strategy,” they will liaise with the medical communication companies that provide the services of a medical writer. In the past, most of these articles have appeared in the medical literature as case studies detailing some physician's experience with the drug. The articles then become part of the sales force strategy, expanding the database of available publications and strengthening the position of their drugs against those of competitors. Acting in this manner, the pharmaceutical companies have distorted the profile of the drugs in the medical literature by selecting only the reports of those physicians who have had a positive experience with the drug for their publications strategy and neglecting all the negative responses.

### Reprints and Pharmaceutical Advertising Profits

The ghostwritten articles are bought in great quantities by the pharmaceutical company's marketing division for distribution by their sales representatives and then appear in their promotional materials as if the ghostwritten articles were independent verification of the efficacy and safety of their drug (McHenry, 2005). In the case of an approved indication, the reprints are distributed with promotional packages and ‘Dear Doctor’ letters. With unapproved indications, the reprints find their way into what are called ‘Med Query Letters,’ which is a legal loophole for off-label promotion. A single reprint can cost as much as $836,000 and net the journal $450,000 in a high profit margin (Smith, 2006). Reprint profits, however, account for a relatively small sum compared to the revenue from pharmaceutical advertising, which typically falls within the range of $715,000 to $18,000,000 (Glassman *et al*., 1999; Gottlieb, 2006). Glassman *et al*. discuss limitations in their study due to the fact that journal staff uniformly rebuffed queries about business matters – a point confirmed by the present author's experience when attempting to gain access to information about conflict of interest policy, circulation rates, and advertising revenue.

While a few voices complain that this creates a dangerous dependence on pharmaceutical revenue and compromises the accuracy of the literature, many journals have turned a blind eye to the infiltration by adopting ‘soft’ or ‘passive’ conflict of interest policies. Medical journal editors with their own conflicts of interest simply violate their own policies rather than offend their pharmaceutical clients (Lexchin and Light, 2006). The editor of the *Lancet* reports that he was routinely pressurized to publish more favourable views of the pharmaceutical industry (Horton, 2004).

### Truth (or the Lack Thereof) and Consequences

Ghostwriting is objectionable to the extent that it deceives readers of the journals into believing that they are reading the work of independent researchers who are named as authors of the papers. In the topsy-turvy world of medical writing, it is standard practice for authors to become “editors” and editors to become “authors.” This would not be so alarming, however, if the results of research were reported accurately. The medical writers who produce the manuscripts are trained in the shift from a “data-driven pursuit” to a “message-driven model” in producing a paper that ensures that commercial relevance is balanced with scientific credibility (Fugh-Berman, 2005). Websites for medical communication companies boast of their ability to create and sustain awareness for a concept, drug, or device, ensuring success in the market.

Medical writing has contributed enormously to distorted profiles of drugs or medical devices because the sponsor companies effectively control the manuscript. Named authors and investigators at the sites of clinical trials seldom see the raw data from all sites reported in the final paper because the company that sponsored the trial owns the data. It is only after the company's research and development and legal departments have signed off on the manuscript that it is released to the “lead author” for submission to the journal. Favourable reports in the publications are guaranteed by withholding any adverse efficacy and safety results and by the very design of the trial, which escapes detection by the peer-review process. Comparing the trial drug with a treatment known to be inferior, testing it against too low a dose of the competitor drug, excluding placebo responders in the washout phase of the trial, or using multiple endpoints in the protocol in order to select for publication the ones that produce favourable results are all common strategies to ensure success (Smith, 2005; Berger, 2002; Berger *et al*., 2003; also see Krimsky, 2006).

Authors who submit manuscripts critical of these practices or those who seek to call attention to scientific misconduct in the clinical trials will routinely find that their papers are rejected for mysterious and arbitrarily *ad hoc* reasons. As Fava remarks in this connection: “Investigators who swim against the tide of corporate-driven research strategies may indeed have difficulty in publishing their findings and observations. If the dialogue in clinical science is censored, the development of new paradigms is hampered” (Fava, 2004). On occasion, manuscripts will be accepted and then rejected at the time of publication on the basis of escape clauses in contracts or on the advice of legal counsel to the journal. In the worst cases, what one is permitted to say is restricted by the journal's assessment of the risks of potential legal action by the pharmaceutical companies, especially in the United Kingdom where libel laws have evolved to protect power and privilege. The cases of David Healy, Nancy Olivieri, and Aubrey Blumsohn have shown the consequences to medical careers for those who refuse to read the results of research in the manner prescribed by the sponsor companies (Healy, 2004; Schafer, 2004; Baty, 2005).

There are of course exceptions to this disturbing trend and some progress is being made toward addressing the problems. Editors such as Richard Smith, formerly of the *British Medical Journal*, have fought the infiltration. Some medical organizations such as Healthy Skepticism, No Free Lunch, Social Audit, The Prescription Project, International Committee of Medical Journal Editors (ICMJE), and World Association of Medical Editors (WAME) have attempted to protect the integrity of medical research by exposing scientific misconduct and formulating conflict of interest policies; but the vast organization and powerful lobby of pharmaceutical marketing is currently winning against scientific accuracy.

While the cases of Merck's Vioxx, Pfizer's Celebrex, GlaxoSmithKline's Paxil/Seroxat, and Avandia and Lilly's Zyprexa have received media attention for a failure of regulation and misconduct in the reporting of data, the majority of other cases largely disappear from the public consciousness because they are settled out of court and the misdeeds disappear as part of the terms of the settlement (see Angell, 2006).

Lilly's Xigris and Bayer's Trasylol, two lesser-known but relatively recent cases, reveal how marketing hype and manipulation of information usurped scientific objectivity and led to serious harm (Singh and Singh, 2007a, 2007b). All of this is unfortunately typical of the fierce industry competition that has resulted in a race to the ethical bottom. While justice might be served for clients, there is enormous disservice to the public good. This means that we all become guinea pigs in the post-marketing surveillance, given the failure to convey honestly the results of the research that brings the drugs to the market. While it would seem that rigorous testing of their drugs would be in the company's best, long-term interest, as long as the corporate structure is driven by marketing rather than science, there is very little hope that there will be any deviation from the goal of maximizing the value of their shareholders′ stock. Even the probability of expensive litigation is factored into the cost-benefit analysis of bringing a new drug into the market.

## The Relevance of Popper's Critical Rationalism

### Control and Suppression of Research

There is little doubt that Popper would view the pharmaceutical industry as an enemy of the open society. He argued, first, that rigorous science had to put itself at a risk of being demonstrated false, i.e., falsifiability of hypotheses and, second, that this practice had to be protected from those influences that would impede scientific progress. Confirmations, for Popper, are relatively easy to obtain and especially so if industry is in control of the process of testing, but a confirmation should only count if it is the result of a genuine attempt at falsification. Protecting the hypotheses by *ad hoc* modifications or by designing experiments that make them immune to refutation always lowers the scientific status of the hypotheses or puts them in the same category as pseudoscience (Popper, 1963). With uncanny vision into our current situation, he writes:

How could we arrest scientific and industrial progress? By closing down or by controlling, laboratories for research, by suppressing or controlling scientific periodicals and other means of discussion, by suppressing scientific congresses and conferences, by suppressing universities and other schools, by suppressing books, the printing press, writing and, in the end, speaking. All these things which indeed might be suppressed (or controlled) are social institutions. Language is a social institution without which scientific progress is unthinkable, since without it there can be neither science nor a growing and progressive tradition. Writing is a social institution and so are the other organizations for printing and publishing and all the other institutional instruments of scientific method. Scientific method itself has social aspects. Science, and more especially scientific progress, are the results not of isolated efforts but of the free competition of thought. For science needs ever more competition between hypotheses and ever more rigorous tests. And the competing hypotheses need personal representation, as it were: they need advocates, they need a jury and even a public. This personal representation must be institutionally organized if we wish to ensure that it works. And these institutions have to be paid for and protected by law. Ultimately, progress depends very largely on political factors; on political institutions that safeguard the freedom of thought: on democracy (Popper, 1961).

This has proved to be prophetic, namely, the *suppression* and *control* of the scientific process by industry that we have today. Pharmaceutical companies largely *control* the research agenda, i.e., what gets done as research and what gets reported in the journals and they *suppress* what is contrary to their constricted self-interest. When knowledge is viewed as the intellectual property of the industry that has sponsored the research, we have nothing but the marketplace as the test. Yet it is clear that the marketplace has generally failed to expose the extent of the corruption or to reveal the flaws in medicines fast enough to protect patients from harm. Industry is not programmed to do critical, scientific testing; rather it is designed to circumvent the process, to minimize financial loss, eliminate competition, and suppress criticism.

Regarding Popper's last point, the present situation in academic medicine points to a failure of government to regulate and, more specifically, a failure to protect scientific objectivity from commercial forces. The free market cannot trump the interests of the open society in scientific progress. But the pharmaceutical industry's influence on medicine is one of the greatest obstacles to this goal, of which our political institutions appear to have no interest in protecting, especially when the interests of medicine conflict with the interests of industry. In this regard, the open, democratic society has become an oligarchy of corporations whose interests serve the profit motive of industry and shape public policy, including weakened regulative bodies such as the FDA in the United States and the MHRA is the United Kingdom.

### Problem Sources and Their Solutions

Marcia Angell, former editor-in-chief of *The New England Journal of Medicine*, has confronted these issues directly. In a chapter entitled “Buying Influence – How the Industry Makes Sure It Gets Its Way,” she exposes the influence of pharmaceutical lobbying on government and the political networks that maintain the status quo (Angell, 2004). In the United States, the pharmaceutical industry is the largest lobby in Washington D.C. As Angell reports on figures from 2002, she found that 675 lobbyists for the pharmaceutical industry are employed full-time at a cost of $91 million to lobby less than half that number of members of Congress. PhRMA, the industry's trade organization, accounted for $14 million of the lobbying expenditures and 112 of the lobbyists (Angell, 2004). This explains why industry-friendly legislation, such as the Bayh-Dole Act of 1980, passes through Congress unopposed – the main piece of legislation in the United States that accelerated the crisis of credibility (Horton, 2005). This need not detract from the positive contribution of the Act to spur entrepreneurship in biomedicine, but it does not condone the unregulated corporatization of medicine.

Aside from a complete political revolution, there are no easy answers. One way to counter this trend is to raise awareness regarding the issues so that all stakeholders look to their enlightened self-interest (Singh and Singh, 2007). And until they do, they should be pressured relentlessly – by raising ethical concerns, by exposure in the lay media, and by lawsuits – so that research integrity prevails and the scientific record is protected.

Angell's prescription for the ethical maladies of medicine and politics includes strengthening of the FDA, the creation of an institute to oversee clinical testing of drugs, and prohibition of pharma-sponsored medical education, all of which would aid in protecting the integrity of medical research. What is noticeably absent from her recommendations is a measure to tackle the most serious problem: the infiltration of medical literature. Since it has become clear that self-regulation against conflicts of interest is a failure for the reasons outlined above, there must be a mechanism for the adoption and enforcement of uniform standards (Dresser, 2006). At present, organizations such as WAME have no powers to enforce uniform policies against scientific misconduct. They review cases submitted for evaluation, but do not pursue an investigation into the facts of the case. Instead, they offer a general opinion after reviewing available materials, but their recommendations are anonymous and not specific to an author or sponsoring company. In the United States, the National Institute of Health (NIH) has an Office of Research Integrity, but it is only pertinent to misconduct in NIH-funded grants. WAME recommends that complaints be directed to the universities in which researchers have been guilty of violating policy. This seldom happens, since university administrations are unlikely to pursue complaints against researchers who bring in as much as $10 million per year in clinical trial research. The key opinion leaders are often presented as model professors at their institutions.

Popper is right that ultimately progress depends on political institutions that safeguard freedom of thought. In the present situation, this must begin with a ban on monetary contributions to our political leaders from the pharmaceutical industry. In addition to Angell's recommendations, I would propose that WAME or ICMJE adopt a censure list for medical journals found in violation of established policy, especially for those with soft conflicts of interest policy. Editors can demand to know whether the clinical trials are registered, whether there was a contract with a medical communications firm, and whether the investigators actually wrote the manuscript and had access to the raw data. The journals can publish retractions of the previously published articles that misrepresent the data. Transparency is also necessary in the business dealings of each journal with regard to advertising revenue and reprint profits. Those that fail to do so should lose the aegis of WAME or ICMJE in much the same manner in which censure functions in defending academic freedom in organizations such as the American Association of University Professors. This will only be possible if office bearers of such organizations are from journals whose policies and practices are themselves above censure.

Journals can regularly invite, rather than suppress, critical evaluation of articles reporting results of industry-sponsored clinical trials. If the critical evaluations reveal that the results of the trial are biased in favour of the study medication, the concerned pharmaceutical company should be put on notice that future study results will be subject to the same rigorous scrutiny. The pharmaceutical companies cannot be permitted to withhold the clinical trial data that allows for the very possibility of critical evaluation. Compulsory trial registration is one means of exposing the failure to report negative trial results, but it is not in itself sufficient. Any industry-sponsored trial submitted for publication cannot be taken at face value without an independent analysis of the data – a genuine attempt at Popperian falsification. And this can take place only if a there is a detailed, critical analysis of the original protocol against the final published paper, since much of the manipulation occurs in the production of the manuscript (Chen *et al*., 2007).

Of course, criticism is not profitable. Pharmaceutical companies will not order reprints or fly authors to conferences to present the results of their evaluation. But, following Popper, it is essential for intellectual advance, academic freedom, and for restoring the profession's confidence in medical literature.

## Concluding Remarks

1. Academic freedom is seriously curtailed in academic medicine due to the obtrusive influence of the pharmaceutical industry.
2. The crucial role of critical reasoning is suppressed due to the protection of commercially valuable content in medical articles favourable to the interests of industry.
3. As such, the pretense of open, free inquiry of democratic society has been supplanted by an oligarchy of corporations whose interests dominate medical societies and medical journal content.
4. Strict policies that protect critical inquiry and academic freedom are required to reverse this trend and keep in check the results of clinical research.

### Take Home Message

Pharmaceutical companies have become the patrons of medicine, but this patronage comes at a high price. Academic freedom cannot exist in a discipline in which industry controls the research agenda and the dissemination of data. It is therefore imperative that medical science wins back its autonomy and restores confidence in its literature.

## Questions That This Paper Raises

1. How has the commercialisation of medicine curtailed academic freedom?
2. What is the impact of pharmaceutical funding on the content of medical journals?
3. How are editors of medical journals influenced by pharmaceutical funding?
4. Do corporate interests compromise the goals of an open, free, democratic society?
5. What is the value of critical reasoning in maintaining an open, free, democratic society?
6. What are the solutions offered to address these concerns?

## About the Author

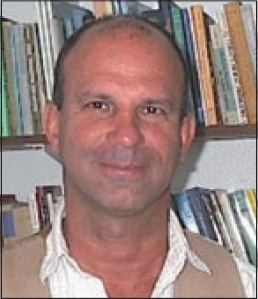



Leemon McHenry read philosophy for his PhD at the University of Edinburgh, Scotland. He currently divides his time between teaching logic, ethics, and philosophy of science at California State University, Northridge, and doing research in medical ethics for the Baum Hedlund law firm.
